# An ensemble of AHP-EW and AE-RNN for food safety risk early warning

**DOI:** 10.1371/journal.pone.0284144

**Published:** 2023-04-26

**Authors:** Jie Zhong, Lei Sun, Enguang Zuo, Cheng Chen, Chen Chen, Huiti Jiang, Hua Li, Xiaoyi Lv

**Affiliations:** 1 College of Software, Xinjiang University, Urumqi, China; 2 College of Information Science and Engineering, Xinjiang University, Urumqi, China; 3 Xinjiang Uygur Autonomous Region Product Quality Supervision and Inspection Research Institute, Urumqi, China; 4 College of Architecture and Urban Planning, Tongji University, Shanghai, China; 5 Xianyang Science and Technology Bureau, Xianyang, China; Sichuan Agricultural University, CHINA

## Abstract

Food safety problems are becoming increasingly severe in modern society, and establishing an accurate food safety risk warning and analysis model is of positive significance in avoiding food safety accidents. We propose an algorithmic framework that integrates the analytic hierarchy process based on the entropy weight (AHP-EW) and the autoencoder-recurrent neural network (AE-RNN). Specifically, the AHP-EW method is first used to obtain the weight percentages of each detection index. The comprehensive risk value of the product samples is obtained by weighted summation with the detection data, which is used as the expected output of the AE-RNN network. The AE-RNN network is constructed to predict the comprehensive risk value of unknown products. The detailed risk analysis and control measures are taken based on the risk value. We applied this method to the detection data of a dairy product brand in China for example validation. Compared with the performance of 3 models of the back propagation algorithm (BP), the long short-term memory network (LSTM), and the LSTM based on the attention mechanism (LSTM-Attention), the AE-RNN model has a shorter convergence time, predicts data more accurately. The root mean square error (RMSE) of experimental data is only 0.0018, proving that the model is feasible in practice and helps improve the food safety supervision system in China to avoid food safety incidents.

## Introduction

Food safety is an essential indicator in evaluating the development of a country’s livelihood. In recent years, food contamination incidents have occurred frequently, and the crisis of public confidence in food safety has become more and more serious. Large-scale food contaminants will cause serious public health events, pose a significant threat to people’s health, and have far-reaching effects on society and the economy [1]. According to WHO, the 31 foodborne hazards counted in 2010 alone resulted in about 600 million cases of illness, 420,000 deaths, and 33 million disabilities [2]. In addition, the lack of nutritional elements in food affects the health of the national public. In China, food safety incidents have also occurred frequently, such as the melamine milk powder incident [3], the “clenbuterol” food poisoning incident [4], and the gutter oil incident [5]. Frequent food safety accidents have exposed the defects in China’s food safety inspection system [6]. In addition to the fact that China’s food regulatory foundation has always been relatively weak compared to developed countries, China’s food safety regulatory system urgently needs reform.

In order to prevent the recurrence of food safety incidents, supervision departments should use scientific data analysis tools for risk assessment and prediction to ensure food quality [7]. We propose a food safety risk early warning model combining the analytic hierarchy process based on the entropy weight (AHP-EW) with the autoencoder-recurrent neural network (AE-RNN). It can check the contaminant and nutrient factors affecting food safety and accurately predict comprehensive food risk values. The main contributions are as follows.

1. This paper proposes an AHP-EW combined with AE-RNN for food safety early warning method to achieve risk prediction and early warning analysis efficiently and rapidly. In this paper, we adopt an improved hierarchical analysis process to obtain the risk value using a data-driven approach to information fusion of testing indexes, eliminating the human intervention of expert evaluation. According to the characteristics of massive and high-dimensional food testing data, the AE-RNN model is established for accurate risk prediction. Overall, intelligent end-to-end risk prediction and early warning of food testing data are achieved.
2. Quantitative analysis of the risk of qualified products and the defining of food safety warning thresholds for qualified products. For food testing data, the regulatory authorities determine whether the test samples are unqualified according to national standards is only the minimum standard. Qualified products also have certain risks. In this paper, we integrate the testing data of contaminant and nutrient indexes to quantify the risk of testing samples. By defining the risk threshold to classify qualified samples, it is beneficial for manufacturers and regulatory authorities to take appropriate measures to minimize the probability of food safety accidents.
3. A large and sufficient experimental validation with real data sets shows that our method is more advantageous. Our model is compared with the mainstream machine learning model for risk prediction on the actual testing data of dairy products in a Chinese enterprise. The experimental results show that the error of data prediction of this paper model is reduced from 0.009 to 0.0018, and the time cost is reduced from 244s to 14s, which proves that this paper model can efficiently and accurately screen out the testing samples with food safety problems in dairy products.

Our model can help supervision departments quickly screen out unqualified samples, trace the root cause of high-risk foods’ existence, guide manufacturers to standardize production management correctly [8], and urge them to formulate risk mitigation strategies [9]. It effectively reduces the occurrence of food safety incidents and safeguards the food safety of Chinese society.

## Related work

The basis for ensuring food safety is a scientific and reasonable risk analysis based on food detection data. Early researchers tried to use mathematical statistics, informatics and other methods to fully explore the intrinsic characteristics and connections of food detection data, such as control chart analysis [10], association rule mining [11], and signal analysis [12]. However, these methods can only make predictions on the state of food safety and cannot quantify specific risks.

The evaluation and early warning methods for food safety risk are continuously developed to conduct detailed risk analysis on food data, such as Bayesian modeling based on mathematical statistics [13], fuzzy integrated evaluation method [14], gray correlation analysis method [15], and artificial neural network-based methods. Specifically, Bouzembrak and Marvin used a constructed Bayesian model to classify food fraud reports and predict the type of food fraud [13]. Shen et al. proposed a comprehensive risk assessment model for import and export food by combining the rough set theory with the conventional fuzzy comprehensive assessment method [14]. Lin et al. proposed an improved interpretive structure modeling (ISM) method based on gray correlation analysis (GRA), which performs a hierarchical analysis of factors affecting food safety [15].

However, all statistical modeling methods rely on expert experience and lack objectivity. Moreover, food detection data are characterized by high dimensionality and complexity, and the regression prediction effect based on statistical analysis is unstable. In contrast, the artificial neural network (ANN), a parallel distributed information processing structure with flexible and fast learning algorithms, diverse network topologies and high fault tolerance, is a powerful tool for data analysis [16]. Based on the potential ability of ANNs to efficiently and accurately process complex data, many scholars have applied related networks to predict food safety risks. Zhang et al. constructed a food safety early warning model based on back propagation (BP) neural network and conducted preliminary validation [17]. However, their work could only make an overall risk assessment on monthly food data and could not be precise to individual samples. Moreover, the BP algorithm converged slowly, and as an optimization method of local search, its training process was prone to fall into local optimal value [18]. Mao et al. proposed a credit evaluation system in the food supply chain based on a combination of long short-term memory (LSTM) network and blockchain to assess the food safety quality status [19]. However, this system can only predict the “negative” and “positive” food safety status and lacks deeper risk analysis. Geng et al. proposed a neural network based on the deep radial basis function (RBF) to build a food risk warning model [20]. They then combined the LSTM network with the method of data fuzzy transformation to fuse the data of various food detection indexes to obtain the comprehensive risk value [21]. However, the two models have their defects. For the RBF network, the k-means method randomly determines the original cluster center, making the clustering results vary with the initial position and easily fall into local optimal solutions. The data fuzzification algorithm used by the latter can not accurately locate a high-risk sample due to the setting of the data interval. Multiple test samples must be checked for screening, which brings unnecessary trouble to the later risk positioning and wastes regulatory resources. Moreover, the performance of LSTM is still a thorny problem for a more considerable amount of data or longer sequences.

In contrast, we use autoencoders for pre-training deep neural networks [22] to obtain an efficient representation of the data, which makes up for the shortcomings of previously used ANN models in handling complex data. The RNN differs from standard neural networks such as the BP and the RBF. It allows the output of the hidden layer neurons to be fed back as input to the neurons. In this way, the RNN has a memory function [23]. Compared with BP and RBF, RNN can find the sequence relationship between samples, so it is the first choice model to deal with serial data. The food detection data used in this paper has the characteristics of time series. That is, the data change is continuous over some time. The data is not independent, and the data of a particular period will impact future data. Therefore, RNN can show better prediction performance for food inspection data trained in time series, addressing the shortcomings of insufficient generalization ability caused by easy falling into the optimal local solution of the above model.

As an objective method for assigning weighting factors, entropy-based methods use information entropy to determine the dispersion of indicators and calculate weights and have been widely used in many fields, such as uncertainty measure [24], safety evaluation [25], and decision problems [26]. We combine the entropy weight method and analytic hierarchy process to obtain the comprehensive risk value of food inspection data. This method not only overcomes the shortcomings of the analytic hierarchy process that does not have an objective basis [27], but also makes up for the shortcomings of the previous algorithm that cannot precisely locate high-risk samples resulting in wasted regulatory resources.

Based on the above, we obtain the comprehensive risk values of food inspection data through AHP-EW, use AE-RNN to achieve feature extraction and conduct two-stage regression training. It compensates for the defects of the previously used models in complex data processing and falling into local optimal solutions and obtains more accurate risk prediction results.

## Integrated food safety risk warning system based on APH-EW fusion AE-RNN

### Problem statement

Given food detection data *X* ∈ *R*^*n*×*m*^ wherein *n* is the number of test samples and *m* is the number of indexes, the objective is to learn an evaluation function *f*(⋅) according to a batch of detection sample so as to calculate the comprehensive risk value *y*_*i*_ = *f*(*x*_*i*_) of a new sample. The comprehensive risk value *y*_*i*_ represents the possibility of food safety problems in the sample *x*_*i*_. The unqualified samples and the qualified samples with higher risk can be screened out through the set unqualified risk threshold and the high-risk threshold [28].

### System overview

**Fig 1:**
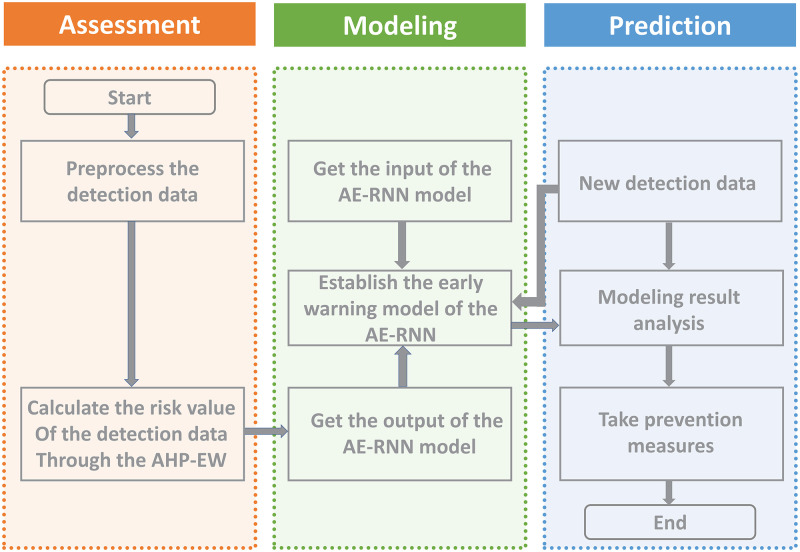
The flow chart of the food safety risk warning system.

The flow chart of the food safety risk pre-warning and control system combing APH-EW with AE-RNN is shown in Fig 1. This risk pre-warning and control system is divided into risk assessment, model construction and risk prediction.

**Fig 2:**
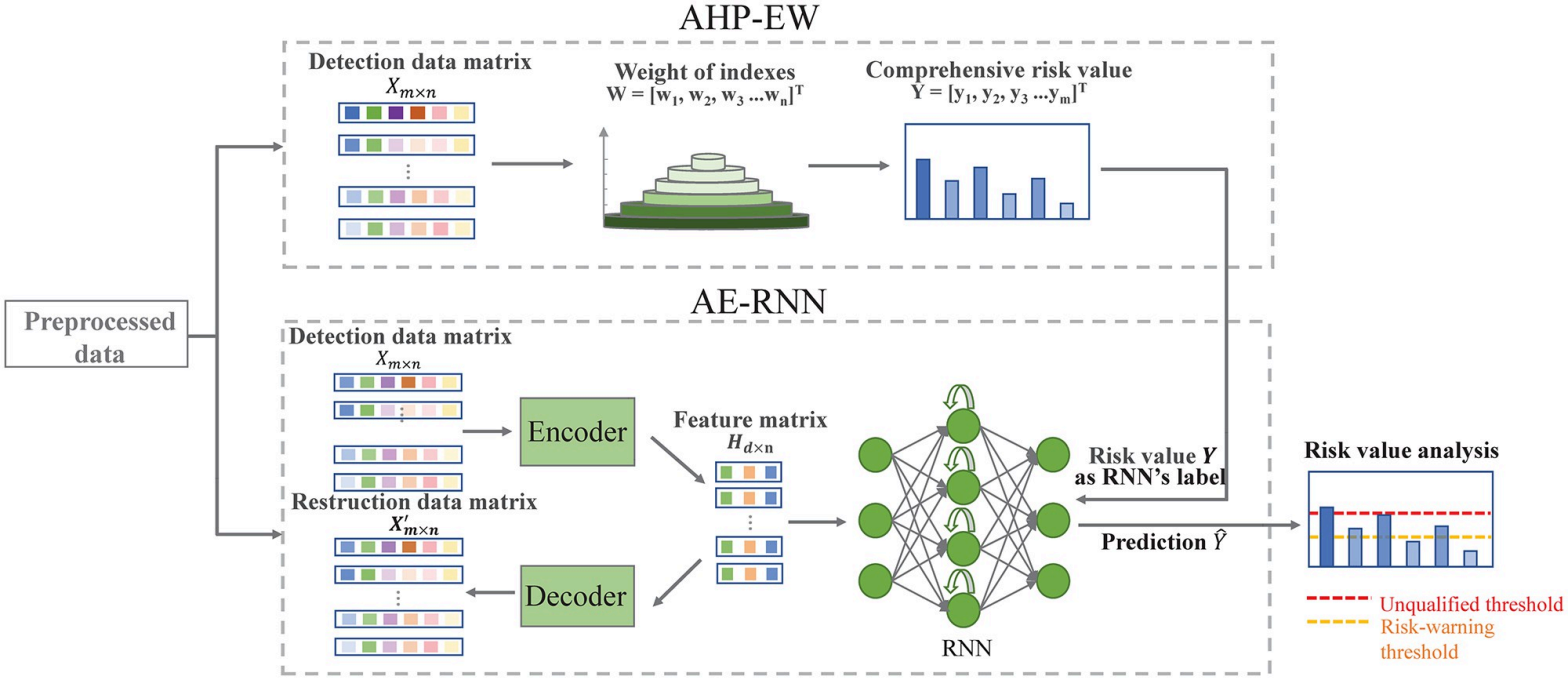
The integrated flow chart including both AHP-EW and AE-RNN methods.

Firstly, the detection data is preprocessed in the risk assessment. The weight of each risk index is calculated by the AHP-EW algorithm, which is multiplied and added with the detection data to obtain the comprehensive risk value of the detection sample as the expected output value of AE-RNN. Secondly, the detection data are divided into training and testing sets in the model construction. The network parameters of AE-RNN are debugged for network training. The AE-RNN model is established, and its prediction performance is evaluated compared to the baseline models. Finally, in the risk prediction, the AE-RNN risk prediction model is used for the risk prediction of new testing samples. The integrated flow chart including both AHP-EW and AE-RNN methods is shown in Fig 2. The threshold is determined by the initial risk value obtained from the risk assessment session. The unqualified samples and qualified samples exceeding the threshold of high-risk value are screened out and reported to the supervision departments, which facilitate regulatory authorities to carry out further risk analysis, trace the specific unqualified indexes and take timely regulatory and preventive measures.

### Data preprocessing

According to the technical requirements of the national food safety standard for sterilized milk (GB 25190–2010), the raw material requirements, sensory requirements, physical and chemical indexes, contaminant limits, mycotoxin limits and microbial limits of dairy products need to meet the content and standards specified by the state. However, the test results of each index are characterized by complexity and different types of data, where the descriptive text cannot even be quantified [29]. We selected lactose, acidity, non-fat milk solids, fat, protein and aflatoxin M1 as the risk evaluation indexes to ensure the validity of the test data in risk prediction. Due to the test data’s different dimensions and prescribed limits for each index, we normalized the test data into dimensionless data to avoid interference with calculating comprehensive risk value [30].

As lactose and aflatoxin have maximum limits, i.e., positive indexes, the higher the value of the index, the greater the potential food safety risk. Non-fat milk solids, fat, and protein indexes have minimum limits, i.e., negative indexes. The lower the detection value, the greater the potential food safety risk. Acidity is limited by interval value, i.e., an interval index. The potential risk is greater if the value is too high or low. For the characteristics of the above indexes, we designed the following data preprocessing methods, as shown in Table 1. In previous studies, interval limit indexes like acidity were categorized as positive or negative for data preprocessing. We propose a pioneering data preprocessing algorithm for interval indexes to quantify it more scientifically and obtain reasonable comprehensive risk values for practical risk evaluation.

**Table 1:** Data preprocessing methods for various types of indexes.

Index category	Index name	Preprocessing method
Positive index	Lactose, Aflatoxin M1	$x_{mn}^{\prime }=\frac{x_{mn}-x_{n}^{\text{min}}}{x_{n}^{\text{max}}-x_{n}^{\text{min}}}$
Negative index	Non-fat milk solids, Protein	$x_{mn}^{\prime }=1-\frac{x_{mn}-x_{n}^{\text{min}}}{x_{n}^{\text{max}}-x_{n}^{\text{min}}}=\frac{x_{n}^{\text{max}}-x_{mn}}{x_{n}^{\text{max}}-x_{n}^{\text{min}}}$
Interval index	Acidity	$x_{mn}^{\prime }=\frac{\left|x_{mn}-x_{n}^{\text{mean}}\right|}{x_{n}^{\text{max}}-x_{n}^{\text{min}}}$

Wherein, *x*_*mn*_ is the detection value of the nth index in the *m*th sample, $x_{n}^{\text{min}}$, $x_{n}^{\text{max}}$, $x_{n}^{\text{mean}}$ are the minimum value, the maximum value and the average value of the nth index in all samples, respectively. It unifies the numerical dimension of each index and eliminates the influence of different data dimensions on the comprehensive risk value by the preprocessing methods.

### The analytic hierarchy process based on the entropy weight

The analytic hierarchy process is a multi-criteria decision-making method widely used in resource allocation and conflict resolution [31]. However, this method mainly judges the relative importance of each index based on experts’ personal experience, which lacks a certain degree of objectivity. Inspired by the work of [32], this paper adopts the combination of entropy weight method and analytic hierarchy process, takes the value of the data itself as the objective basis, and fuses each index through AHP-EW to obtain a comprehensive risk value.

For the *m*th sample in the input data, the standard correlation function *f*_*mn*_(*x*) of its *n*th index is obtained from Eq (1).

$$\begin{array}{c} \begin{array}{c} f_{mn}\left(x\right)=\{\begin{array}{cc} 0 & x\notin [x_{n}\left(1\right),x_{n}\left(3\right)]\\ \frac{x_{mn}-x_{n}\left(1\right)}{x_{n}\left(2\right)-x_{n}\left(1\right)} & x\in [x_{n}\left(1\right),x_{n}\left(2\right)]\\ \frac{x_{n}\left(3\right)-x_{mn}}{x_{n}\left(3\right)-x_{n}\left(2\right)} & x\in [x_{n}\left(2\right),x_{n}\left(3\right)] \end{array}\\ m=1,2,\ldots ,p;n=1,2,\ldots ,q \end{array} \end{array}$$ (1)

Wherein, *x*_*n*_(1), *x*_*n*_(2) and *x*_*n*_(3) are the minimum, average, and maximum values of the *n*th index, respectively.

Assuming that the data after preprocessing is *X* = [*X*(1), *X*(2), *X*(3), …, *X*(*m*)], we obtain the matrix *U*_*m*×*n*_ using the standard correlation function described above.

$$\begin{array}{c} U_{m\times n}=[\begin{array}{cccc} u_{11} & u_{12} & \ldots & u_{1n}\\ u_{21} & u_{22} & \ldots & u_{2n}\\ \cdots & \cdots & \cdots & \cdots \\ u_{m1} & u_{m2} & \cdots & u_{mn} \end{array}] \end{array}$$ (2)

Normalize each value of the matrix *U*_*m*×*n*_.

$$\begin{array}{c} u_{mn}^{\prime }=\left(u_{mn}-\bar{u_{n}}\right)/S_{n}\left(m=1,2,\ldots ,p;n=1,2,\ldots ,q\right) \end{array}$$ (3)

Wherein, $\bar{u_{n}}=\frac{1}{p}\sum_{m=1}^{p}u_{mn}\left(n=1,2,\ldots ,q\right)$, $S_{n}=\sqrt{\frac{1}{p-1}\sum_{m=1}^{p}{\left(u_{mn}-\bar{u_{n}}\right)}^{2}}\left(n=1,2,\ldots ,q\right)$.

Then the negative numbers in the matrix are changed to positive numbers by the formula $v_{mn}=u_{mn}^{\prime }-t_{n}+\varepsilon$, wherein, $t_{n}=min\left(u_{mn}^{\prime }\right)<0\;\left(n=1,2,...,q\right)$.

The positive matrix *V*_*m*×*n*_ is obtained from the above.

$$\begin{array}{c} V_{m\times n}=[\begin{array}{cccc} v_{11} & v_{12} & \ldots & v_{1n}\\ v_{21} & v_{22} & \ldots & v_{2n}\\ \cdots & \cdots & \cdots & \cdots \\ v_{m1} & v_{m2} & \cdots & v_{mn} \end{array}] \end{array}$$ (4)

The m-dimensional symmetric matrix is obtained from Eq (5).

$$\begin{array}{c} D=VV^{T}=[\begin{array}{cccc} d_{11} & d_{12} & \ldots & d_{1m}\\ d_{21} & d_{22} & \ldots & d_{2m}\\ \cdots & \cdots & \cdots & \cdots \\ d_{m1} & d_{m2} & \cdots & d_{mm} \end{array}] \end{array}$$ (5)

The entropy represents the amount of information provided by the data. The basic idea of the entropy weight method is to determine the weight based on the variability of the index. Generally speaking, the larger the differences among the values of an index, the lower the value of information entropy, which suggests that the index provides a higher amount of useful information, which renders a higher weight to the index [33]. The *e*_*m*_ of each sample was obtained from Eq (6).

$$\begin{array}{c} e_{m}=-\frac{1}{\text{ln}\;p}\sum_{n=1}^{q}\left(d_{mn}\;\text{ln}\;d_{mn}\right)\left(m=1,2,\ldots ,p\right) \end{array}$$ (6)

The data weight *h*_*m*_ for each sample was obtained from Eq (7) [34].

$$\begin{array}{c} h_{m}=\frac{1-e_{m}}{\sum_{m=1}^{p}\left(1-e_{m}\right)}\left(m=1,2,\ldots ,p\right) \end{array}$$ (7)

The information value of each index is obtained from Eq (8).

$$\begin{array}{c} c=X^{T}h \end{array}$$ (8)

$$\begin{array}{c} w_{q}=\frac{c}{\sum_{n=1}^{q}c} \end{array}$$ (9)

The weight vector is *W* = [*w*_1_, *w*_2_, *w*_3_ …*w*_*q*_]^*T*^, and the fused data *Y* is obtained from Eq (10).

$$\begin{array}{c} Y={\left[\begin{array}{c} y_{1}\\ y_{2}\\ \cdots \\ y_{p} \end{array}\right]}^{T}=XW=[\begin{array}{cccc} x_{11} & x_{12} & \cdots & x_{1q}\\ x_{11} & x_{22} & \cdots & x_{2q}\\ \cdots & \cdots & \cdots & \cdots \\ x_{p1} & x_{p2} & \cdots & x_{pq} \end{array}][\begin{array}{c} w_{1}\\ w_{2}\\ \cdots \\ w_{q} \end{array}] \end{array}$$ (10)

**Algorithm 1** AHP-EW

**Require:** The test data *X*

**Ensure:** The fused data *Y*

1: Preprocess the data *X* = [*X*(1) *X*(2) *X*(3) …*X*(*m*)];

2: Calculate the matrix *U*_*m*×*n*_ using the standard correlation function *f*_*mn*_(*x*) via Eq (1);

3: **for** *m* = 1 : *p* **do**

4:  **for** *n* = 1 : *q* **do**

5:   $u_{mn}^{\prime }=\left(u_{mn}-\bar{u_{n}}\right)/S_{n}$ via Eq (3);

6:   $v_{mn}=u_{mn}^{\prime }-t_{n}+\varepsilon$;

7:  **end for**

8: **end for**

9: matrix *D*←*VV*^*T*^ via Eq (5);

10: **for** *m* = 1 : *p* **do**

11:  *e*_*m*_←$-\frac{1}{\text{ln}\;p}\sum_{n=1}^{q}\left(d_{mn}\;\text{ln}\;d_{mn}\right)$, *h*_*m*_←$\frac{1-e_{m}}{\sum_{m=1}^{p}\left(1-e_{m}\right)}$ via Eqs (6) and (7);

12: **end for**

13: *c*←*X*^*T*^*h* via Eq (8);

14:*w*_*q*_←$\frac{c}{\sum_{n=1}^{q}c}$ via in Eq (9);

15: *Y*←*XW* via in Eq (10);

16: Produce the fused data $Y=[y_{1},y_{2},\ldots ,y_{q}]$;

## Construction of AE-RNN model

### Autoencoder

**Fig 3:**
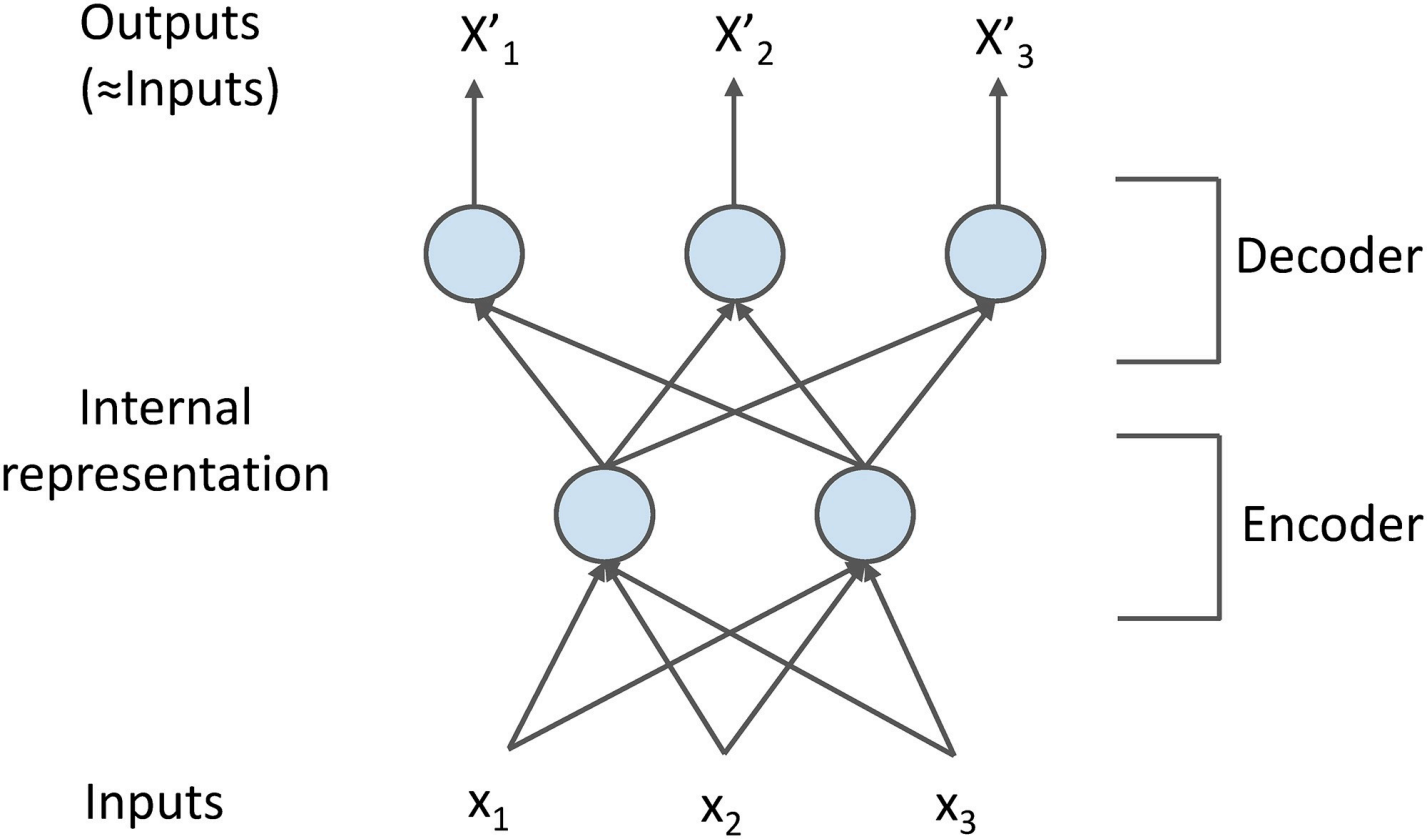
The autoencoder structure.

Autoencoder consists of an encoder and a decoder, which is an unsupervised neural network including three layers: input layer, hidden layer and output layer, as shown in Fig 3. Its input data and output data have the same dimension. The output data should reproduce the input data to the maximum extent to maintain the consistency of input and output by optimizing the objective function [35]. It is assumed that each input data sample with *m* variables is represented as *x* = [*x*_1_, *x*_2_, …, *x*_*m*_]^*T*^ ∈ ***R***^*m*^, and the output data sample is reconstructed as $x^{\prime }={\left[x_{1}^{\prime },x_{2}^{\prime },\ldots ,x_{m}^{\prime }\right]}^{T}\in R^{m}$.

The encoder maps the input data *x* to the hidden layer by Eq (11) to obtain the hidden features *h*.

$$\begin{array}{c} h=\sigma_{1}(W_{1}x+b_{1}) \end{array}$$ (11)

The decoder reconstructs the output *x*′ based on Eq (12) using the hidden feature *h*.

$$\begin{array}{c} x^{\prime }=\sigma_{2}(W_{2}h+b_{2}) \end{array}$$ (12)

As an unsupervised learning framework, autoencoder is usually used for dimension reduction or feature extraction. By limiting the dimension of the hidden layer features *h*, the autoencoder can be forced to capture the most salient features in the training data and obtain the valuable features of the input data [36]. Since autoencoder can make feature extraction, we superimpose it on a deep learning model. The overall model uses salient data features to mine the underlying patterns of the data and achieve more accurate data predictions.

### Recurrent neural network

**Fig 4:**
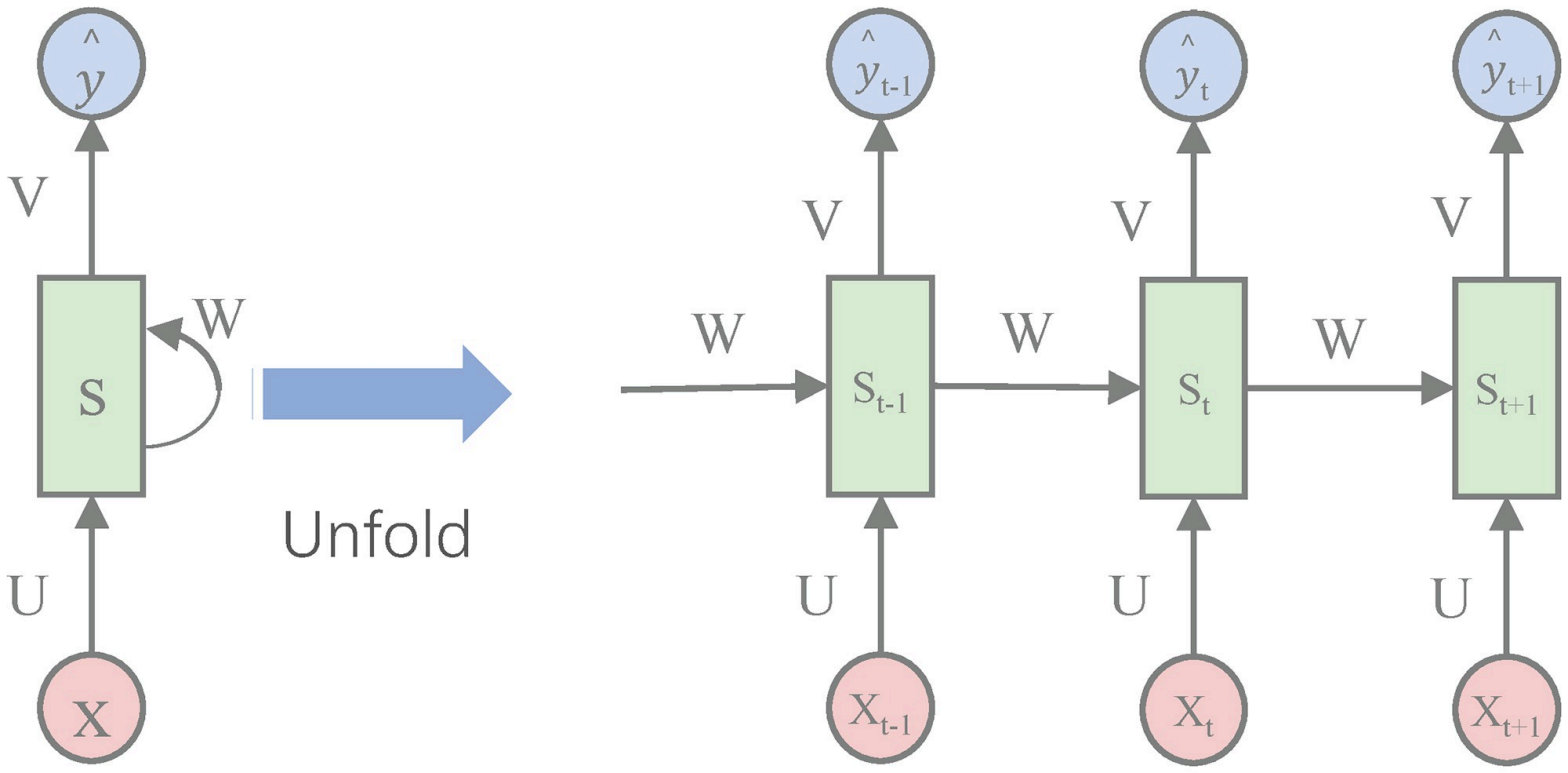
The RNN structure.

The traditional neural network model is fully connected from the input layer to the hidden layer and then to the output layer, with no connection between the nodes in each layer. The current output of a sequence in the RNN is related to the previous outputs, as shown in Fig 4. The network memorizes and applies the previous information to calculate the current output. The nodes between hidden layers are connected, and the input of the hidden layer includes not only the output of the input layer but also the output of the hidden layer at the last moment [37].

*X*_*t*_ denotes the input at step *t*, and *S*_*t*_ is the state of the hidden layer at step *t*, which is the memory unit of the network. *S*_*t*_ is calculated based on the output of the current input layer with the state of the hidden layer at the previous step [38].

$$\begin{array}{c} S_{t}=f(U\cdot X_{t}+W\cdot S_{t-1}) \end{array}$$ (13)

Wherein, *U* is the weight matrix from the input layer to the hidden layer, and *W* is the weights matrix from the hidden layer at the last moment to the hidden layer at the current moment. It can be seen that the value of *S*_*t*_ depends not only on *X*_*t*_ but also on *S*_*t*−1_.

The value of the output layer is ${\hat{y}}_{t}=g(V\cdot S_{t})$, where *V* is the weight matrix from the hidden layer to the output layer.

### AE-RNN

**Fig 5:**
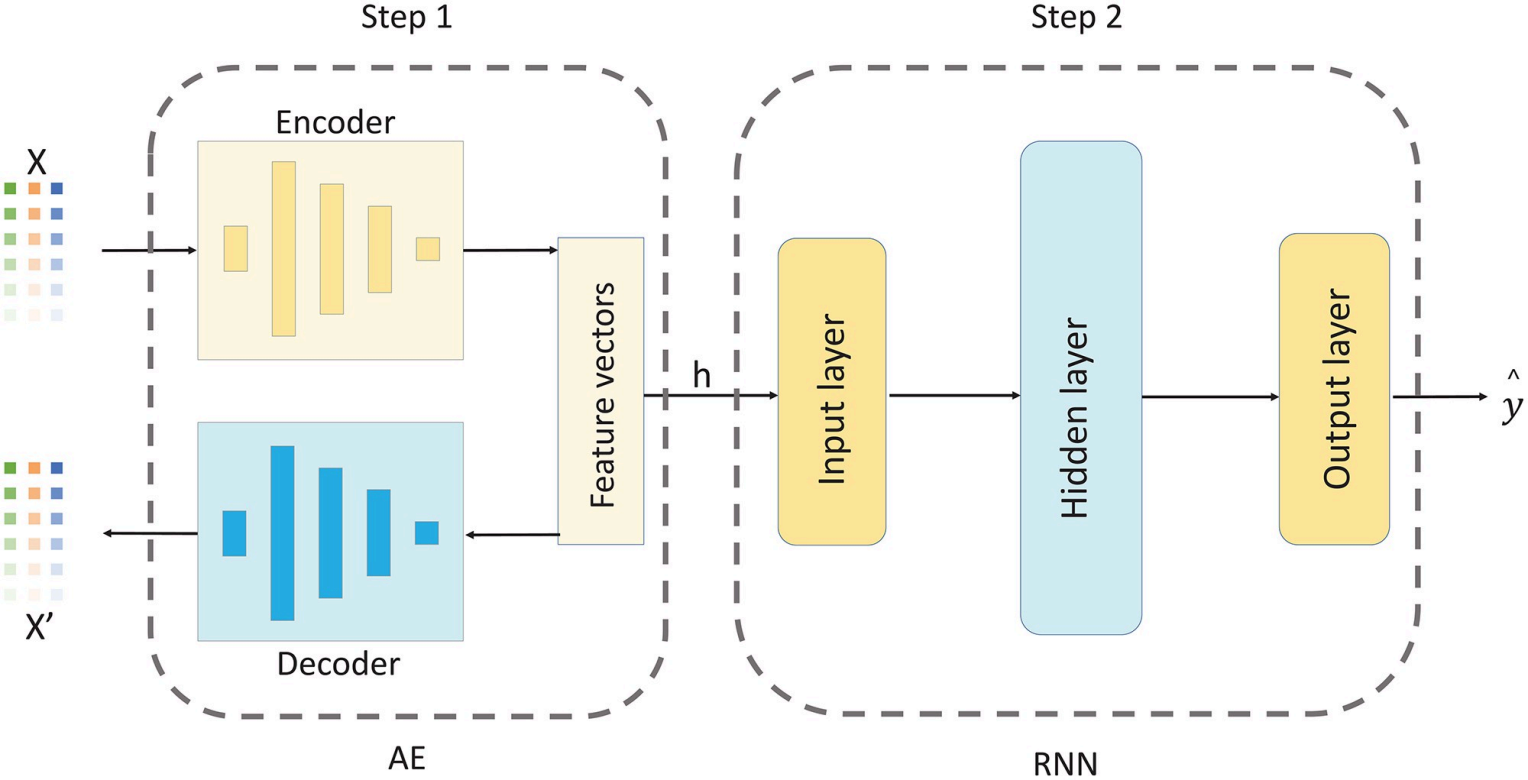
The AE-RNN general structure.

Considering that supervised learning is required to predict food safety risks, the detection model consists of two neural networks, the autoencoder and the RNN, as shown in Fig 5. The training is carried out in two stages. Specifically, in the first stage, the detection data *x* is input to the autoencoder, the hidden layer vector *h* is obtained through the encoder, and then the output value *x*′ is obtained through the decoder. The autoencoder is trained and optimized to reconstruct the input data *x* to the greatest extent from the output data *x*′. The significant feature vector *h* of the input data is obtained by limiting the data dimensionality of the hidden layer to realize the feature extraction work. In the second stage, the feature vector *h* extracted from the autoencoder is fed by the RNN input layer. The input data *h* is nonlinearly transformed by the hidden layer. The output value $\hat{y}$ is obtained by the output layer. The construction and optimization of the RNN model are completed by back propagation of the error between the output value $\hat{y}$ and the expected output *y* to improve the accuracy of risk prediction.

### Risk prediction

After the model’s training, the risk values of unknown products are evaluated using the AE-RNN. By inspecting the risk value of each sample in the risk assessment, we set the thresholds of different risk levels of food testing samples (mainly unqualified and risk-warning samples). The new products are classified into different risk types of samples. Further traceability analysis is conducted for unqualified and risk-warning samples to facilitate the supervision departments taking relevant measures. The detailed analysis and relevant initiatives of the above samples are shown in the case study.

## Case study: Risk warning for dairy products

**Fig 6:**
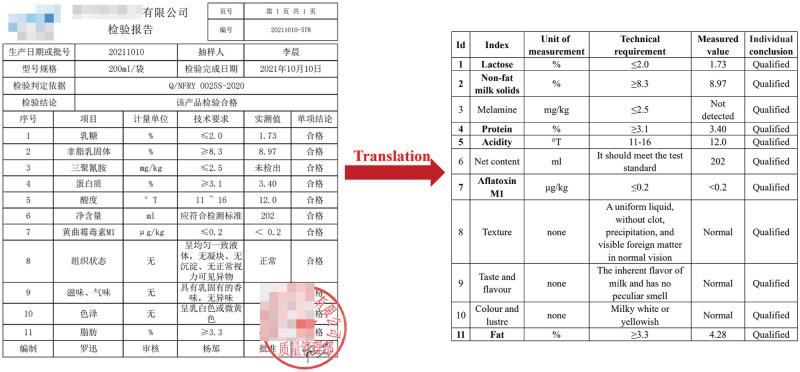
The original detection report(left).

In the case study, we used the self-test data of a dairy brand of sterilized milk in Guizhou Province, which were obtained in strict accordance with the GB 19645–2010 national standard for food safety of sterilized milk following the GB/T 5009 food hygiene test method for selecting specific test indicators, a total of 2017 sets. The technical requirements for sterilized milk are divided into raw material requirements, sensory requirements (color, taste, odor, tissue state), physical and chemical indicators (fat, protein, non-fat milk solids, acidity), contaminant limits, mycotoxin limits, and microbial limits. The data were obtained from the test reports, which have a limited variety of indicators, and the original report is shown in Fig 6.

Since some of the test results cannot be directly applied to the experiment, we have done the following treatment on some data. In order to ensure the authenticity and validity of the data, six valid indicators were retained.

- Delete the sensory quality characteristics of food that are not closely related to food safety, such as taste, color, shape, etc.
- Remove extra symbols. The test result of aflatoxin in the sample was “<0.2”. Since the standard limit of aflatoxin is less than 0.2, we deleted the “<” symbol in the test result and recorded the test value as “0.2”.
- Delete the useless information. Remove all samples of the test results that are “not detected” Melamine indicators.

After processing, each group of test data has 6 indicators, namely lactose, acidity, non-fat milk solids, fat, protein and aflatoxin. The original data format is shown in Table 2. We judge whether each test data meets the national food safety standard for sterilized milk according to the requirements of GB 19645–2010 Hygienic Standard for Sterilized Milk.

**Table 2:** Raw testing data of dairy production.

Id	Lactose	Acidity	Non-fat milk solids	Fat	Protein	Aflatoxin M1
20211010–578	1.73	12.0	8.97	4.28	3.40	<0.2
20211009–516	1.74	12.0	9.12	9.12	3.43	<0.2
20211008–452	1.74	12.0	8.79	8.79	3.42	<0.2
20211007–388	1.72	12.0	9.12	9.12	3.38	<0.2

Firstly, the comprehensive risk values of samples were calculated by the AHP-EW method for the 1987 sets of testing data from September 2016 to September 2021, which were used as the expected output risk value of the AE-RNN network to establish the risk warning model. The model was then used to predict the comprehensive risk value of the 30 testing data sets in October 2021. Finally, the samples with high-risk values in the predicted data are further risk warned and analyzed [29].

**Fig 7:**
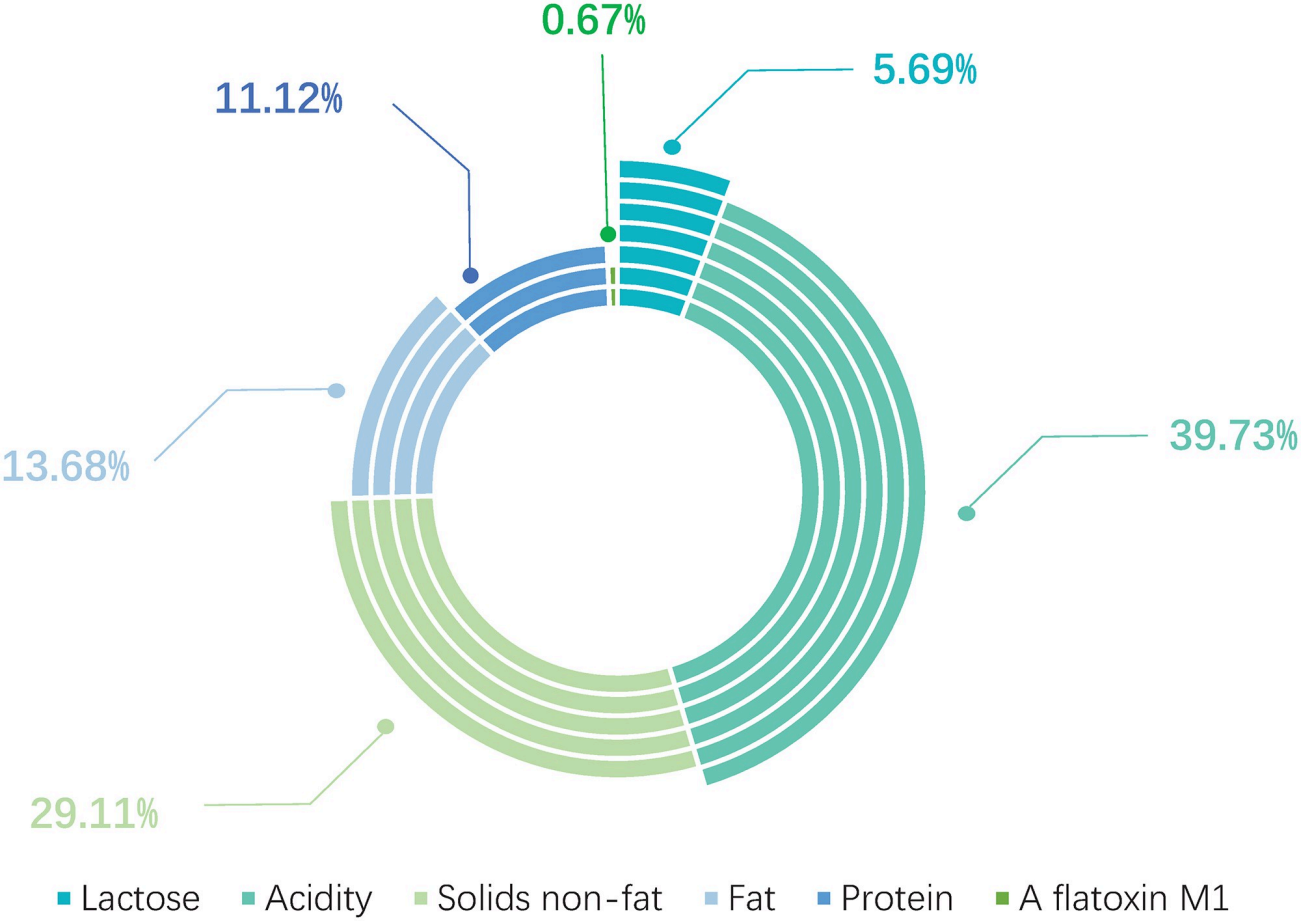
The weighting percentages of detection indexes in dairy products.

We performed data preprocessing for each index with a different limit in the data preprocessing session. We replaced each index value with its difference from the standard value as the data for risk calculation. The greater the difference, the farther the test value is from the standard value. The larger the risk value thus calculated, the greater the probability of disqualification of this test sample, which needs to attract the attention of manufacturers and regulatory authorities. After that, the weight percentages of each index are obtained by AHP-EW, as shown in Fig 7. The preprocessed testing data are multiplied and added with the weights of the corresponding indexes to complete the fusion of the detection indexes, and the comprehensive risk value is obtained. The preprocessed data were used as the input of the early warning model, and the comprehensive risk values were used as the expected output for the training of the AE-RNN risk early warning network.

The BP, the LSTM and the LSTM-Attention were used for model construction for the 1987 training sets, and risk prediction was performed for the 30 sets. The relevant parameters of the four neural network models were set separately. The BP, the LSTM and the LSTM-Attention are three-layer neural network structures with 6 nodes in the input layer, 1 node in the output layer and 16 nodes in the hidden layer. The learning rate is set to 0.001. The BP batch size is set to 8, the epoch is set to 500, and the LSTM and LSTM-Attention batch size is set to 2. The former epoch is set to 30, and the latter epoch is set to 100. The hidden layer nodes of AE-RNN are set to 3, and the 3-dimensional main features are extracted. The learning rate is set to 0.001, and the number of iterations is 1900. In addition, the average relative generalization error (ARGE), root mean square error (RMSE) and model convergence time were used to evaluate the model performance and verify that the AE-RNN model has a better risk prediction effect. The calculation equations are shown in Eqs (14) and (15), wherein *z*_*i*_ is the expected output value and *y*_*i*_ is the actual output value.

$$\begin{array}{c} ARGE=\frac{1}{m}\sum_{i=1}^{m}Abs(\frac{z_{i}-y_{i}}{y_{i}})\times 100\% \end{array}$$ (14)

$$\begin{array}{c} RMSE=\sqrt{\frac{1}{m}\sum_{i=1}^{m}{\left(z_{i}-y_{i}\right)}^{2}} \end{array}$$ (15)

The ARGE and RMSE values for the four prediction models are shown in Table 3. The error values of AE-RNN achieve a multiplicative level improvement compared with the previous three models. It can be concluded that the generalization performance of AE-RNN is better compared with the other three models, and the gap between AE-RNN and the expected risk value is the smallest. The 5-fold cross-validation has verified the fitting data effect of the AE-RNN model. Although the prediction accuracy of AE-RNN has not been effectively improved, it indicates that the model is not over-fitted. The comparison of model training time shows that for the large volume of food inspection data in the regulatory process, the convergence time of BP, LSTM and LSTM-Attention models is too long. The convergence time of the LSTM-Attention model even reaches more than 30 minutes, which lacks practical significance. However, AE-RNN achieves a more accurate prediction effect in only 14 seconds, meeting the demand for accurate and efficient risk prediction in food regulation.

**Table 3:** The comparison of predicted results.

	BP	LSTM-Attention	LSTM	AE-RNN
ARGE	5.11%	4.29%	3.74%	**0.05**%
RMSE	0.016	0.012	0.009	**0.0018**
RMSE(5-fold cross-validation)	0.015	0.013	0.009	**0.0018**
Model convergence time(s)	244	2059	905	**14**

To further validate the generalization of the model, we conducted experiments on two additional data, camel milk and raisin data. We collected 427 camel milk data containing 14 indicators of protein, fat, recovered milk acidity, impurity level, moisture, lead, total arsenic, chromium, nitrite, aflatoxin, total bacterial colony, coliform, Staphylococcus aureus, and melamine according to the local standard DBS65 014–2017 of Xinjiang Uygur Autonomous Region. The reference standard for raisin data is the food safety standard NY-T 705–2003 for seedless raisins, which contains 368 test samples including sulfur dioxide residue, lead, moisture, total acidity, mold, ash, protein, and sodium indicators. The original data and the comprehensive risk assessment values were input into BP, LSTM-Attention, LSTM, and AE-RNN, and the fitting data results and time spent for each model are shown in Tables 4 and 5. AE-RNN still achieved the best-fitting results and took very little time.

**Table 4:** The comparison of predicted results in camel milk dataset.

	BP	LSTM-Attention	LSTM	AE-RNN
ARGE	890.99%	1362.52%	1551.61%	**3.07**%
RMSE	0.05	0.07	0.06	**0.004**
Model convergence time(s)	77	1961	163	**12**

**Table 5:** The comparison of predicted results in raisin dataset.

	BP	LSTM-Attention	LSTM	AE-RNN
ARGE	0.6%	4.4%	0.4%	**0.1**%
RMSE	0.02	0.048	0.017	**0.003**
Model convergence time(s)	41	1670	152	**13**

**Fig 8:**
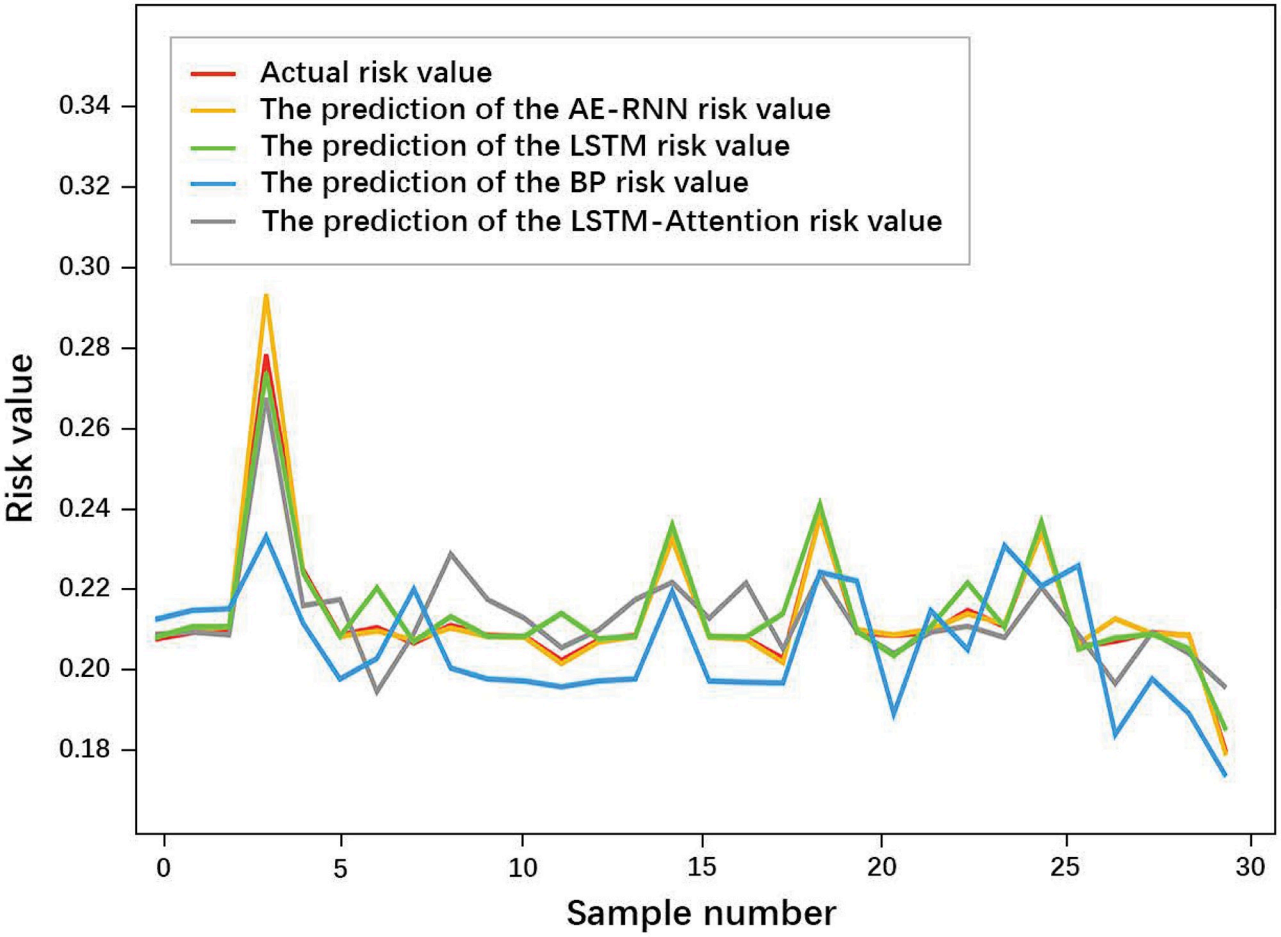
Fitting curves of the four neural networks.

The fitting curves of the BP, the LSTM-Attention, the LSTM, and the AE-RNN are shown in Fig 8. The BP network has the worst data fitting effect and the most significant gap with the expected value. The prediction effect of the LSTM-Attention network is better than the BP network, but there is still a certain gap with the actual value. The prediction performance of the LSTM-Attention and the LSTM is improved in order compared with the BP. However, the AE-RNN has the best data fitting effect. The predicted value curve almost overlaps with the expected value curve on some samples, and the accuracy of numerical prediction is the highest.

**Fig 9:**
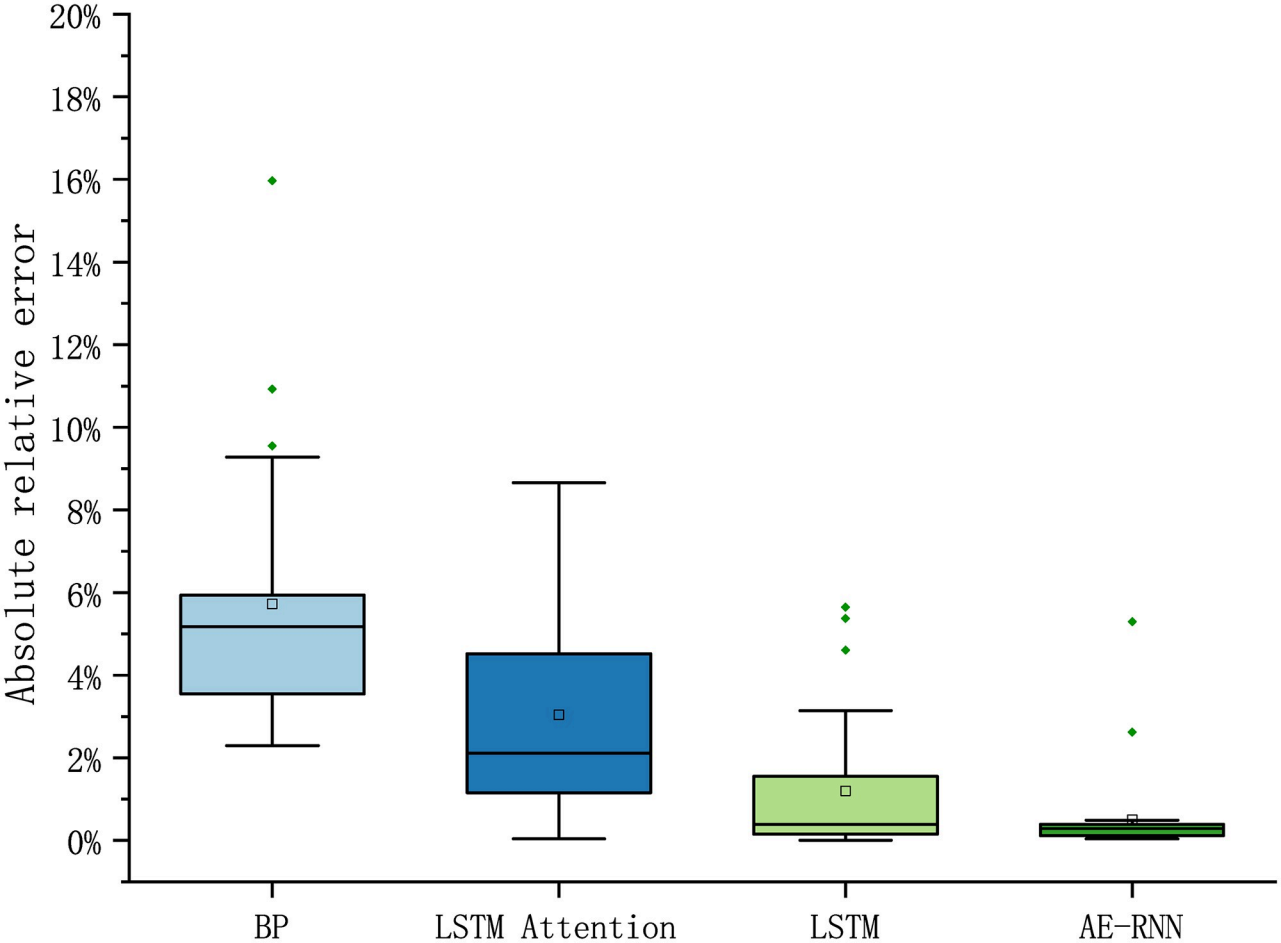
Boxplots of relative error of four neural networks.

The relative error distributions of four neural networks are shown in Fig 9, which shows that more than 50% of the samples in the BP model have prediction errors greater than 5%, and even one sample almost reaches 16%. In the LSTM-Attention model, compared with the BP model, although the error is reduced and 75% of the sample error values are controlled at about 5%, there are still a small number of samples with an error 7%. The relative error of the LSTM model is reduced compared with the previous two. 75% of the samples can be controlled at less than 2%, but the error reaches about 6%. For the AE-RNN, the relative error of more than 75% of the samples was below 1%. It is concluded that the generalization ability of AE-RNN is more robust than that of BP, LSTM-Attention, and LSTM.

We performed ablation experiments for AE and RNN structures, respectively. As shown in Table 6, AE-RNN reduces the fitting time by 4s compared with RNN. The analysis shows that AE achieves the dimensionality reduction of features by feature compression, which improves the efficiency of subsequent model data fitting. RNN has smaller values in two evaluation indexes, ARGE and RMSE, compared with MLP, which indicates that RNN fits the data better and makes more accurate predictions. Therefore, AE-RNN is more efficient and accurate in predicting data than common neural networks.

**Table 6:** The ablation experimental results for AE-RNN.

Architecture	AE	RNN	ARGE	RMSE	Model convergence time(s)
AE-MLP	✓	✕	0.27%	0.0047	13
RNN	✕	✓	0.07%	0.0021	18
AE-RNN	✓	✓	0.05%	0.0018	14

The samples with single or multiple indexes exceeding the national limits are “unqualified” samples. Such samples have food safety hazards of excessive contaminants or nutrient deficiencies, and human consumption will cause health problems. Producers need to destroy the same or related batches of dairy products immediately.

**Fig 10:**
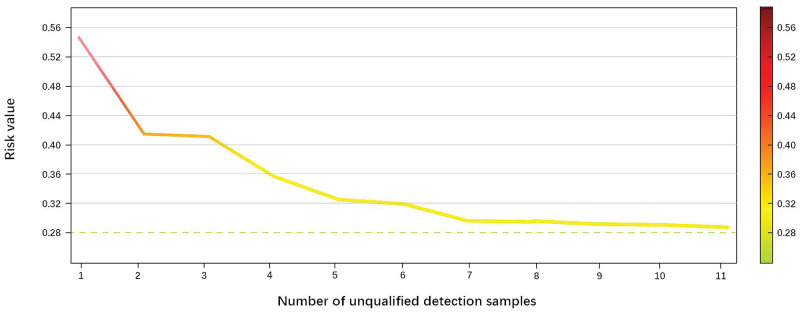
The risk value of unqualified samples from September 2016 to September 2021.

**Fig 11:**
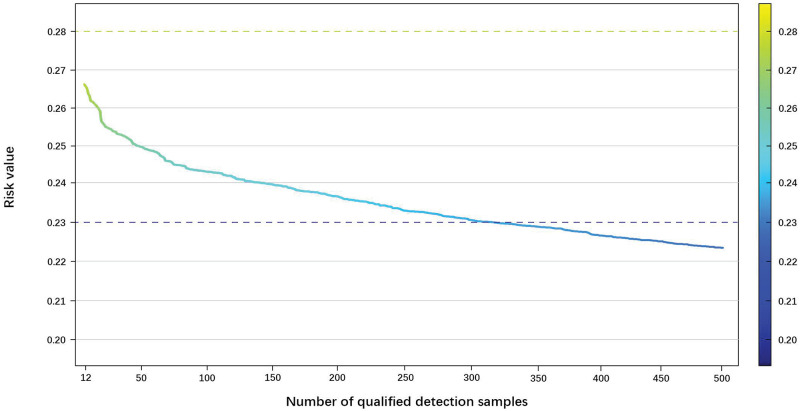
The risk value of part qualified samples from September 2016 to September 2021.

The number of samples that exceeded the limits for single or multiple indexes was statistically obtained as 11 in the 1987 samples. We ranked the risk values obtained by AHP-EW. By verifying the sample IDs, the top 11 samples with the highest risk values were indeed unqualified(Fig 10). The risk value of the 11th sample is 0.281, and the highest risk value of the qualified samples is 0.267(Fig 11). We define the threshold of the unqualified samples as 0.28 based on the rounding principle. Samples with risk values higher than 0.28 are unqualified samples, and samples lower than 0.28 are qualified samples.

**Fig 12:**
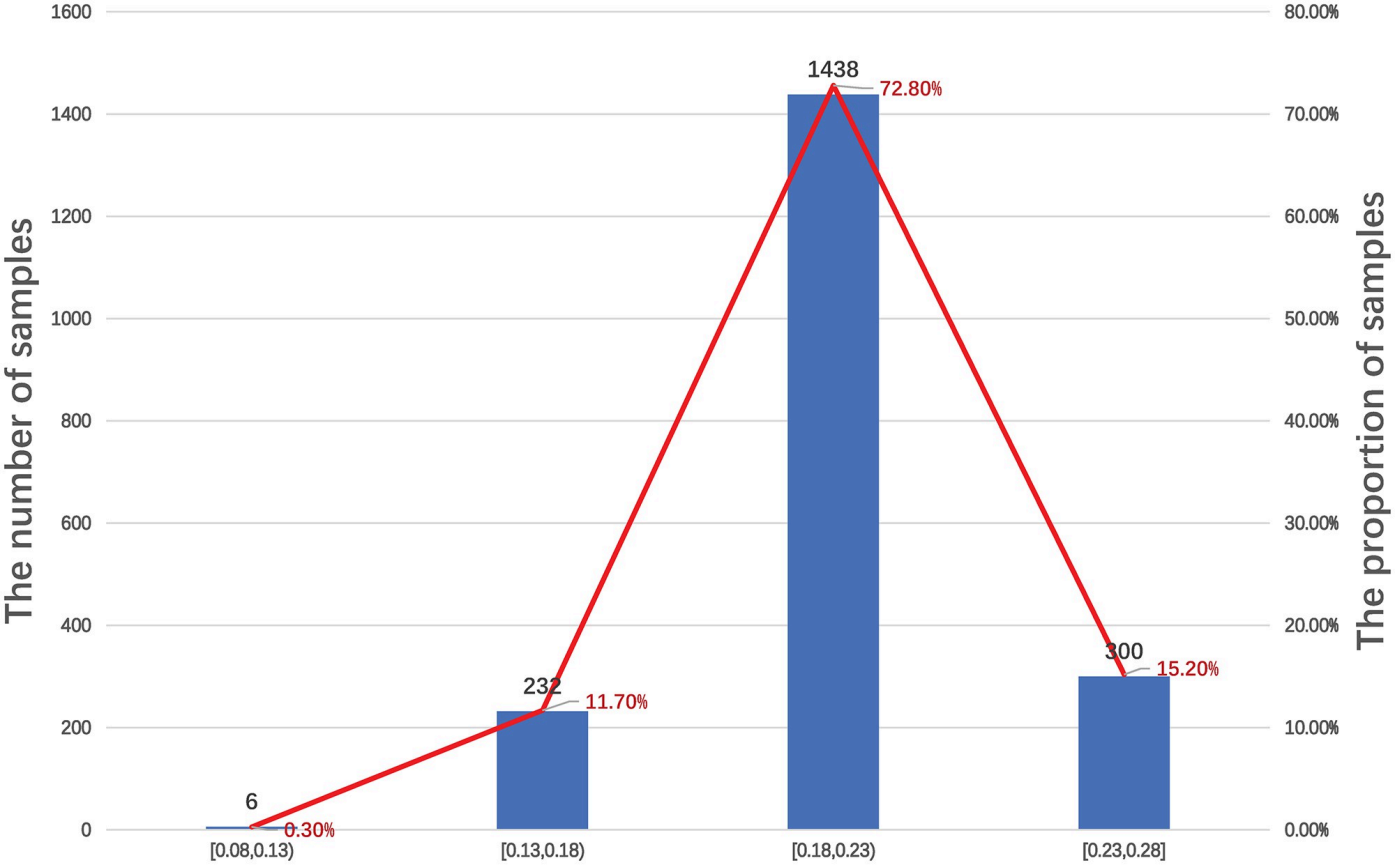
The risk value interval distribution of qualified dairy products.

The goal of risk quantification and early warning is not only to locate unqualified samples but also to identify qualified samples with high risk and safety hazards to draw regulatory authorities’ attention to high-risk samples and minimize the probability of food safety accidents. Although samples with risk values lower than 0.28 are qualified, some samples have a higher risk because one or more indexes are close to the national limits, and we define such samples as “risk-warning” samples. The qualified samples were classified into risk categories based on the distribution of risk values. In order to determine the threshold for “risk-warning” samples, we invited a panel of food safety experts from our collaborators to evaluate the samples in different risk categories to determine whether they should be classified as “risk-warning” samples. The risk range for the qualified samples was [0.08,0.28]. We segmented at 0.05 intervals and classified the qualified samples into four categories: “1”, very low risk, with a value range of [0.08,0.13]; “2”, low risk, with a value range of (0.13,0.18]; “3”, medium risk, with a value range of (0.18,0.23]; “4”, high risk, with a value range of (0.23,0.28]. The distribution of risk value intervals for the qualified samples is shown in Fig 12.

Among the qualified samples, we sequentially put samples into the test set of expert evaluation with a spacing of 50 samples. Finally, we obtained a test set containing two samples of category “1”, five samples of category “2”, 29 samples of category “3”, and six samples of category “4”. Three experts analyze the raw test data from the test set and determine whether the samples need risk warnings based on their expertise and experience. The statistical results of the Food Safety Expert Panel are shown in Table 7.

**Table 7:** Expert judgment statistics.

		Expert A	Expert B	Expert C
Early warning is required	1 [0.08,0.13]	0/2	0/2	0/2
	2 (0.13,0.18]	0/5	0/5	0/5
	3 (0.18,0.23]	1/29	0/29	0/29
	4 (0.23,0.28]	5/6	6/6	6/6

From the statistical results, the Food Safety Expert Panel concluded that the samples requiring early warning were concentrated in the category “4”, i.e., samples with risk values in the range of (0.23,0.28]. Therefore, the threshold for “risk warning” samples was determined to be 0.23, and samples with a risk value greater than 0.23 were considered “risk-warning” samples. Samples with risk values lower than 0.23 are considered low-risk samples.

**Fig 13:**
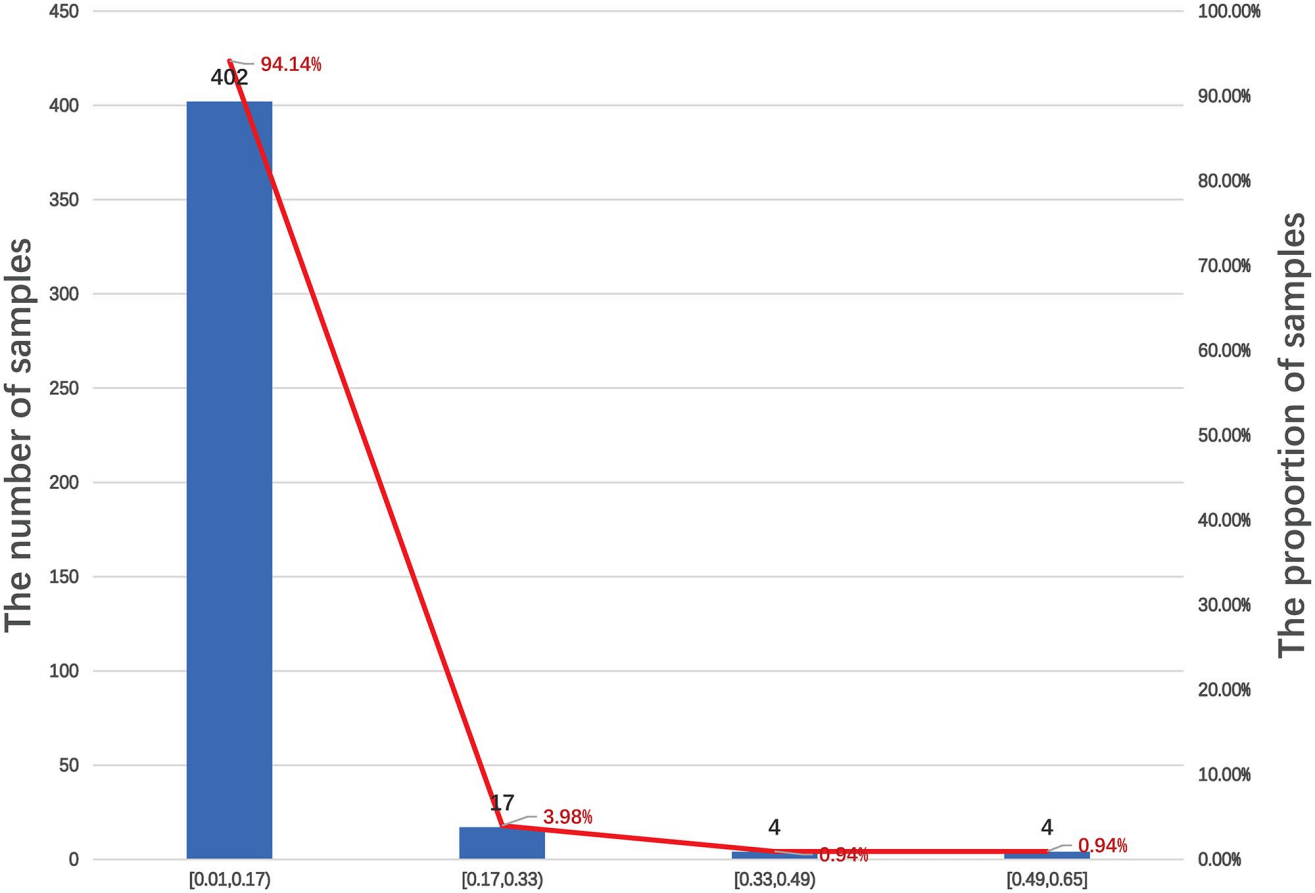
The risk value interval distribution of qualified camel milk.

**Fig 14:**
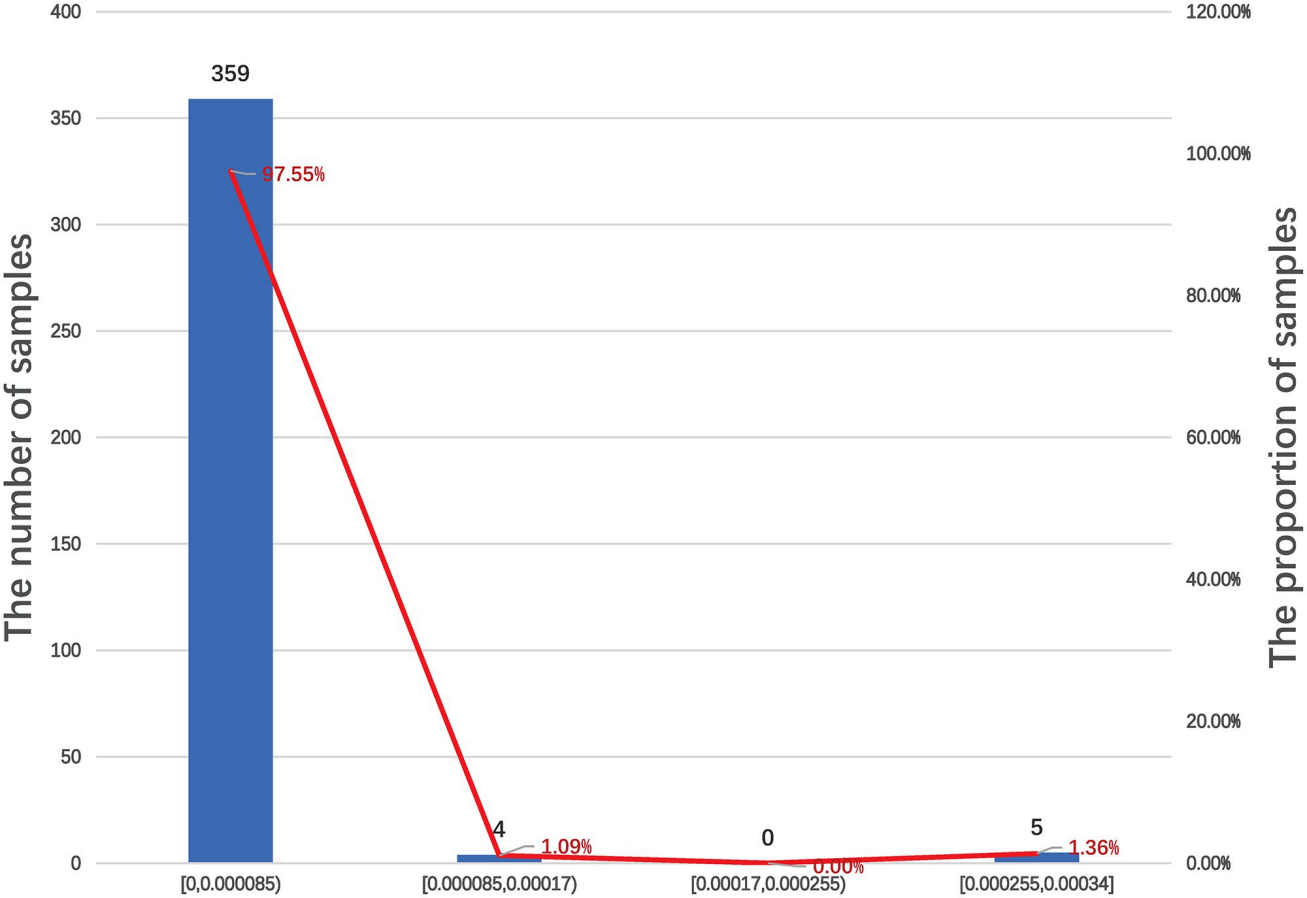
The risk value interval distribution of qualified raisin dataset.

Due to the short time span of the data collection of camel milk and raisins, no unqualified samples were found for the time being, so we only carried out the risk classification of qualified samples. The risk value domain for the camel milk data was in the range of (0.01,0.65), and the risk value domain for the raisin data was distributed in the range of (0,0.0034), and the distribution intervals of the two data are shown in Figs 13 and 14. We determined the “risk warning” sample thresholds for camel milk and raisins as 0.49 and 0.000255, respectively, according to the above process and after the expert review.

**Fig 15:**
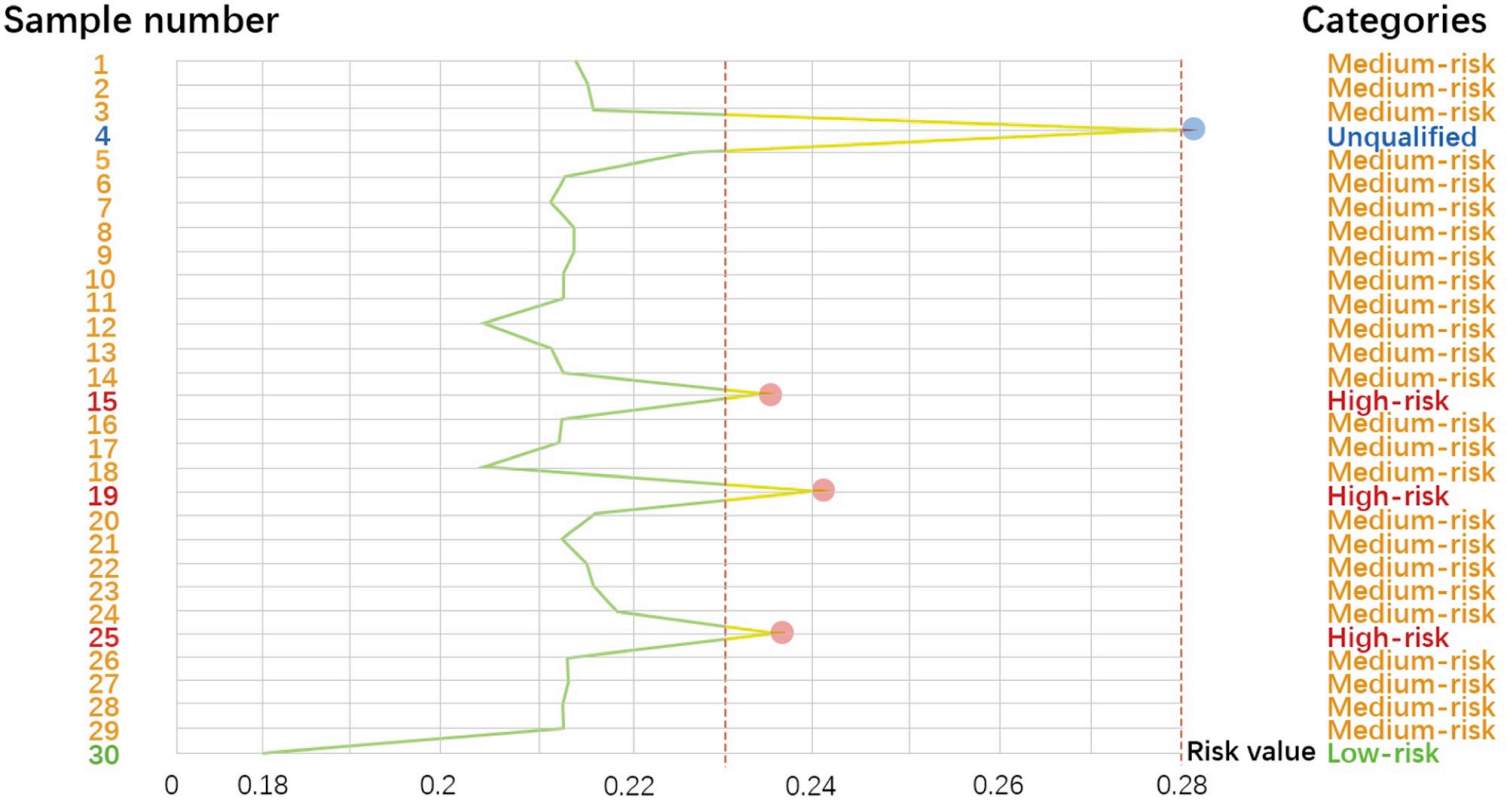
The risk values of samples in October of 2021.

The comprehensive risk value of testing data in October 2021 is shown in Fig 15. The risk value of the fifth sample is 0.281, which has exceeded the threshold value of unqualified samples. The supervision department should immediately urge the manufacturer to interrupt the production process in the production line related to this sample, further analyze the value of the individual index of this sample, and strictly monitor the index in the following production process. The 15th, 19th and 25th high-risk samples are 0.235, 0.24 and 0.237, respectively, which exceeded the established threshold for risk-warning samples. Given the principle of minimizing food safety risk, the supervision department still has to conduct a follow-up risk analysis on relevant samples to identify the individual index that causes high risks and urge manufacturers to control all food production indexes strictly. The risk value of the 30th index is 0.179, which is lower than that of other samples in October 2021, so the safety state is reliable. The risk value of other samples is about 0.21, which does not exceed the early warning threshold, and the food safety state is relatively stable.

After a further risk analysis of the relevant samples, the 5th unqualified sample does not meet the national requirements for non-fat milk solids in sterilized milk. The 15th, 19th and 25th risk-warning samples had lower fat content than others, so the comprehensive risk value is higher. Based on the detailed risk analysis, the supervision departments can pay more attention to these two indexes in this manufacturer and supervise the manufacturer to produce high-quality food in accordance with the national standard content in the production process to avoid food safety incidents.

In addition, we analyzed the potential sources of bias in the experiment. In general, there are three main aspects of potential sources of bias affecting model accuracy [39]: 1. correct identification of statistical objects (data) relevant to the research question. 2. use of representative training data for predictive modeling. 3. selection of representative features to predict the target variable.

First, correctly identify the statistical objects (data) relevant to the research question. Sample selection bias may occur in the sampling process. We conducts random sampling among unencapsulated batches of the same sterilized milk to represent the overall data of this batch. There is no sample selection error caused by the selected specific sample not being fully representative of the whole. In addition, the test data may be noisy and inaccurate. We ensure that the framework accurately learns the overall characteristic distribution of the data through standardization of data preprocessing, increasing the sample size, and final expert review.

Second, representative training data for conducting predictive modeling [40]. Generally, qualified data dominate food testing data, and unqualified data are scarce. In order to avoid the model learning biased patterns using only qualified samples as training data, we use training data with a sample collection period of 6 years, which contains a certain amount of unqualified samples in real situations. The model fully learns the data characteristics of the qualified and unqualified samples.

Finally, representative features were selected to predict the target variables. The indicators we used for dairy products are in the GB 19645–2010 standard. To ensure the authenticity of our data, we included all available indicators in our evaluation system after pre-processing.

In addition, the cost-benefit analysis and practicality of the method in a real environment are important. First, we analyze the time complexity of the model. The AE-RNN is mainly divided into auto-encoder (AE) and Recurrent neural network (RNN). According to *h* = *σ*_1_(*W*_1_*x* + *b*_1_), *x*′ = *σ*_2_(*W*_2_*h* + *b*_2_), where the number of samples, hidden layer neurons and input size are *n*, *m*, *d*, respectively, we can know that the computation process is [*d*, *m*] × [*m*, *n*] + [*d*, *n*] and [*m*, *d*] × [*d*, *n*] + [*m*, *n*], both with complexity O(nmd). According to *h*_*t*_ = *f*(*Ux*_*t*_ + *Wh*_*t*−1_), it is known that the computation process is *Ux*_*t*_: [*d*, *m*] × [*m*, *n*] with the complexity of O(nmd), and *Wh*_*t*−1_: [*d*, *d*] × [*d*, *n*] with the complexity of $O(nd^{2})$, so the computational complexity of the RNN module is $O(nd^{2})$. The time complexity of the whole model that is $O(nd^{2})$. From the above analysis, it is known that the model has low time complexity and has the potential to handle large amounts of data in realistic scenarios.

Further, the experimental results are shown in Tables 3–5. The framework is highly efficient for high-dimensional data (camel milk data) and high-volume data (dairy data). It has an extremely low time cost compared to the current mainstream machine learning models such as BP, LSTM, and LSTM-Attention, which can save about six times the time compared to the optimal model. The data prediction is the most accurate and significantly improves over the optimal prediction of other models. Theoretically, the accurate and fast prediction results are mainly due to the feature compression of AE and the simple network structure and “memory” function of RNN. At the same time, the algorithmic framework is an end-to-end intelligent algorithmic framework. We integrate the AHP-EW algorithm, which requires computational power, into our algorithmic code, and the overall algorithmic model requires a low configuration environment. Users only need a computer and the collected data to obtain the risk values of input samples through the online platform, enabling low-threshold and low-cost food safety risk prediction.

**Fig 16:**
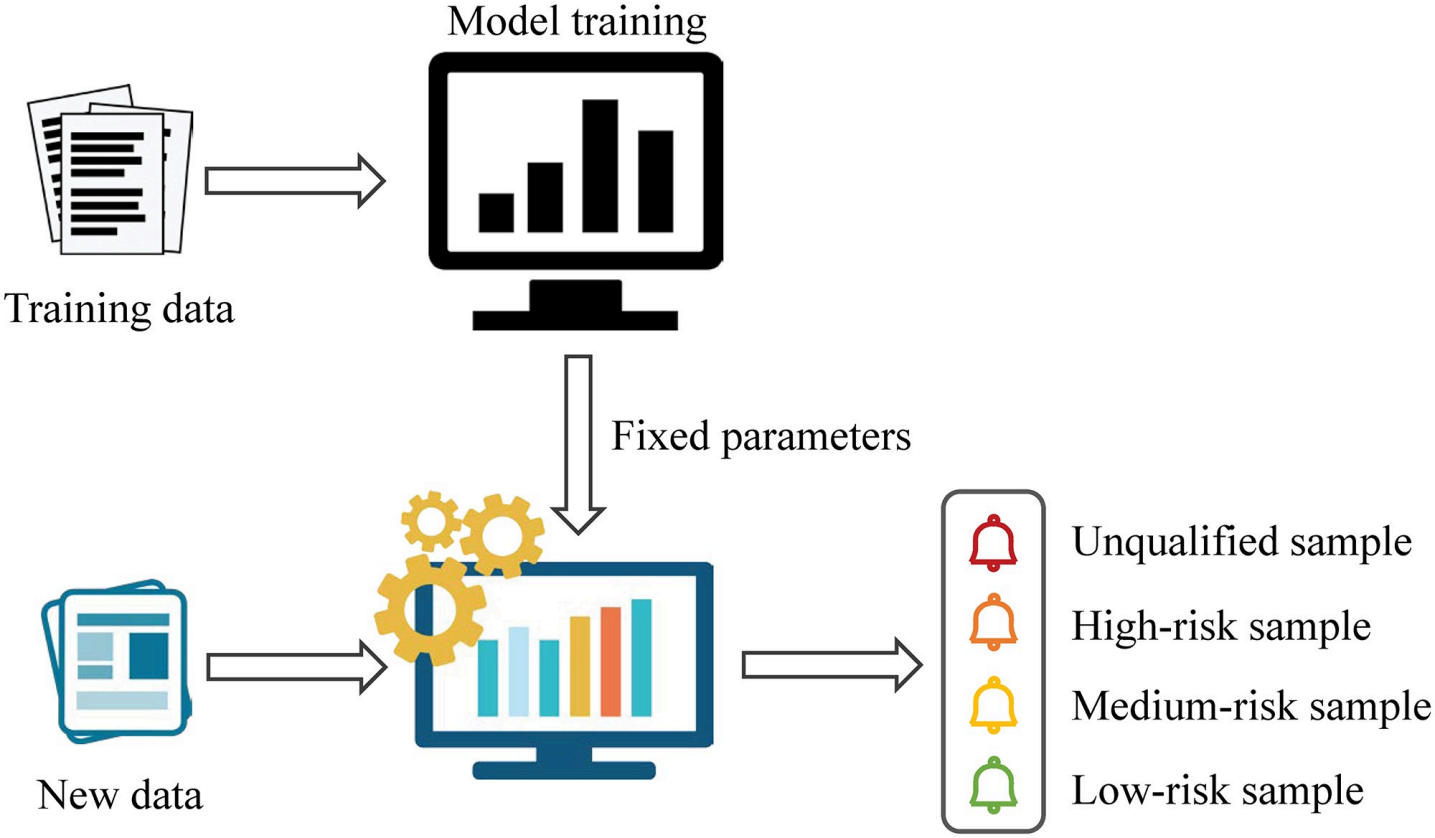
Use process of risk assessment model in real situations.

Finally, we are actively preparing for the launch of this framework in the risk assessment platform of our partner for real scenarios of dairy product testing data. Specifically, the application process of our algorithmic framework is as follows(Fig 16): in the first step, we train the model with relevant detection data and wait for the model to finish data fitting before retaining the network parameters. In the second step, the regulator can upload new detection data to the risk assessment platform, and the data enters the established prediction model to complete a feed-forward. The platform can output the risk prediction value of this batch of data. The regulator can take appropriate measures according to the risk threshold.

## Conclusion

In this paper, a framework for food safety risk pre-warning and control based on AHP-EW and AE-RNN was proposed. This framework was applied to the testing data of sterilized milk of a dairy product brand in Guizhou Province, China. The comprehensive risk values of the product were obtained using the AHP-EW method and used as the expected output of AE-RNN for network construction to achieve risk prediction of the new product. By comparing the three models of the BP, the LSTM and the LSTM-Attention, it is verified that the AE-RNN model has a shorter convergence time, the best data fitting effect and the highest data prediction accuracy.

In the later stage, the supervision departments can further track the high-risk and unqualified samples for detailed risk analysis based on the risk prediction results of this paper’s model and check the single indexes that cause the increase in risk value. The detailed risk analysis will help the relevant departments to take accurate control, urge food manufacturers to make targeted rectifications, and avoid food safety incidents.

In future work, we will explore the processing methods of other data, such as descriptive indexes, expand more indexes that can be included in risk early warning, and make this risk warning model more applicable in the food industry. We will further explore multi-source information fusion methods for risk evaluation, such as evidence theory [41]. In addition, the overall training speed of the RNN model is slow, and the gradient may disappear in training. In this paper, the RNN and the auto encoder are combined to extract features to improve the training speed, and the skills are used in the model training to avoid possible defects. In the future, we will select a more suitable neural network model to achieve better prediction performance.

## Supporting information

S1 File Training data for establishing risk early warning model. A total of 1987 sets of dairy product testing data were collected from September 2016 to September 2021, and the data dimension was 6.
(CSV)

S2 File Test data for risk analysis. 30 sets of data for testing and risk analysis were from October 2021, with a data dimension of 6.
(CSV)

S3 File Camel-milk data. 427 sets of data, with a data dimension of 26.
(CSV)

S4 File Rasine data. 368 sets of data, with a data dimension of 8.
(CSV)
